# Preoperative Serum Albumin-to-Creatinine Ratio Predicts 1-Year Reintervention After Above-Knee Femoropopliteal Bypass Surgery

**DOI:** 10.3390/jcm15124466

**Published:** 2026-06-09

**Authors:** Mert Çelik, Arzu Funda Tarhan, Aykut Şahin, Fatih Enes Durmaz

**Affiliations:** 1 Department of Cardiovascular Surgery, Burdur State Hospital, Burdur 15100, Türkiye; 2 Department of Cardiovascular Surgery, Faculty of Medicine, Eskişehir Osmangazi University, Eskişehir 26040, Türkiye; afundatarhan@gmail.com (A.F.T.); draykutsahin@gmail.com (A.Ş.); 3 Department of Cardiology, Eskişehir City Hospital, Eskişehir 26080, Türkiye; fatihenesdurmaz@gmail.com

**Keywords:** femoropopliteal bypass, peripheral artery disease, serum albumin/creatinine ratio, prognosis

## Abstract

**Objective**: Serum albumin/creatinine ratio (sACR) integrates nutritional–inflammatory status and renal reserve. We evaluated its ability to predict 1-year reintervention for symptomatic graft occlusion in patients undergoing prosthetic above-knee femoropopliteal bypass for peripheral artery disease (PAD). **Methods**: This single-center retrospective study included 132 adults (7 women, 125 men) who underwent Dacron above-knee femoropopliteal bypass. The primary analysis evaluated preoperative sACR as a continuous variable. For descriptive comparisons and Kaplan–Meier visualization, patients were stratified according to the median preoperative sACR value (3.77), yielding two groups: <3.77 vs. ≥3.77. The primary endpoint was reintervention for symptomatic graft occlusion confirmed by imaging. Discrimination was assessed using ROC analysis, and the ROC-derived cutoff was interpreted as an exploratory threshold rather than a validated clinical decision point. Associations with 1-year reintervention were assessed using Cox proportional hazards regression analysis (age, diabetes, hypertension, smoking, coronary artery disease, hemodialysis-dependent chronic kidney disease, hemoglobin level, GFR, total protein level, antiplatelet use, and anticoagulant use). **Results**: Most baseline characteristics were comparable between groups; however, hemodialysis-dependent chronic kidney disease was more frequent in the low-sACR group. Reintervention occurred significantly more often in the low-sACR group from month 1 onward. sACR significantly discriminated 1-year events (AUC = 0.736; *p* < 0.001). The optimal cutoff was ≤3.7 (sensitivity 90.9%, specificity 64.6%). Higher sACR was associated with lower 1-year event risk in both univariate and multivariate models (adjusted HR 0.61, 95% CI 0.43–0.87; *p* = 0.007). **Conclusions**: Preoperative sACR is a practical biomarker associated with early and 1-year reintervention risk after prosthetic above-knee femoropopliteal bypass and may aid perioperative risk stratification.

## 1. Introduction

Peripheral artery disease (PAD) is a major cause of morbidity worldwide and affects more than 200 million people [1]. Its clinical spectrum ranges from asymptomatic patients to critical limb-threatening ischemia (CLTI). The prevalence of peripheral artery disease increases with age, reaching up to 20% in the population over 65 years of age [2].

PAD is not confined to limb-related symptoms; rather, it reflects a systemic atherosclerotic burden associated with substantial cardiovascular morbidity and mortality. Therefore, current guidelines emphasize that controlling risk factors, appropriate treatment strategies, and appropriate revascularization in necessary patients can significantly affect the natural course and clinical outcomes of the disease [3].

Reintervention and clinical presentation in PAD are closely related not only to anatomical factors but also to the patient’s comorbidities, systemic inflammation status, and organ reserve. Chronic kidney disease (CKD) is considered a strong risk factor for the development and progression of PAD [4]. It has also been reported that PAD is more common and has a worse prognosis in the presence of CKD; and that renal dysfunction can also affect the prognosis after revascularization [5].

Serum albumin level is a negative acute phase marker of inflammation and is important because it reflects the nutritional status of the patient, reduces oxidative stress by binding free radicals, and inhibits platelet aggregation [6]. It has been reported that hypoalbuminemia is associated with perioperative morbidity and mortality in patients undergoing lower extremity revascularization; and that low albumin levels may be associated with poor clinical outcomes [7,8].

In recent years, research aimed at predicting the prognosis in peripheral artery disease has focused not only on clinical factors but also on biomarkers. Serum albumin-to-creatinine ratio (sACR) has attracted attention in recent years as a composite parameter that combines the inflammation/nutrition axis of albumin and the renal reserve/comorbidity burden of creatinine in a single measure. The prognostic value of sACR in the cardiovascular disease spectrum has been reported in association with short- and medium-term clinical outcomes in clinical conditions such as acute myocardial infarction, acute coronary syndromes, and heart failure [9,10]. Furthermore, its association with mortality in aortic diseases has been evaluated, and it has been reported that sACR can contribute to risk prediction [11]. However, evidence regarding the role of sACR in predicting clinical outcomes in the PAD population is relatively limited. In this study, we investigated whether preoperative sACR predicts 1-year reintervention after prosthetic above-knee femoropopliteal bypass in patients with PAD.

## 2. Materials and Methods

This single-center retrospective cohort study included adult patients who underwent prosthetic above-knee femoropopliteal bypass with a Dacron graft for PAD between January 2015 and January 2022.

Patients without regular follow-up, those undergoing redo above-knee femoropopliteal bypass, and those operated on for acute limb ischemia were excluded. Preoperative venous blood samples obtained within 24–48 h before surgery were analyzed in the same institutional biochemistry laboratory using standardized automated assays. Serum albumin and creatinine levels were analyzed using a cobas^®^ c 702 module (Roche Diagnostics GmbH, Mannheim, Germany). Serum creatinine was measured using the Jaffe method, and serum albumin was measured using the colorimetric bromocresol green method. eGFR was calculated using the CKD-EPI equation. sACR was calculated by dividing the preoperative serum albumin level (g/dL) by the serum creatinine level (mg/dL), both measured 24–48 h before surgery. Because no established clinical cutoff for sACR exists in patients undergoing prosthetic above-knee femoropopliteal bypass, sACR was primarily analyzed as a continuous variable. Median-based grouping was used only for descriptive comparisons and Kaplan–Meier visualization. Accordingly, patients were stratified according to the median sACR value (3.77) into low-sACR (<3.77; *n* = 66) and high-sACR (≥3.77; *n* = 66) groups for these secondary descriptive analyses.

The primary endpoint was reintervention within 1 year for symptomatic graft occlusion confirmed by duplex ultrasonography and/or computed tomography angiography. Graft occlusion was defined as the complete absence of graft flow detected by duplex ultrasonography and/or computed tomography angiography during follow-up evaluations. Reintervention was defined as the embolectomy procedure performed to restore graft patency due to symptomatic graft obstruction.

Primary patency was defined as uninterrupted graft patency without any surgical reintervention. Primary-assisted patency was not specifically evaluated because prophylactic interventions performed before complete graft occlusion were not systematically recorded due to the retrospective study design.

Secondary patency was defined as the restoration and maintenance of graft patency after surgical reintervention following graft occlusion. Limb salvage was defined as freedom from major amputation above the ankle level, including below-knee and above-knee amputation, during follow-up.

Overall survival was defined as the time from the index procedure to death from any cause. Since reintervention was performed in patients who developed loss of graft patency in the study cohort, freedom from reintervention corresponded to the same clinical event time as loss of primary patency. Therefore, freedom from reintervention was evaluated in parallel with primary patency.

All available imaging studies were independently reviewed by radiologists who were blinded to the patients’ sACR group assignment and clinical outcomes.

Independent Variables of the Study:

Demographic variables were recorded as age and gender. Hypertension, diabetes mellitus, Hemodialysis-dependent chronic kidney disease (was recorded as KDIGO stage 5 CKD requiring renal replacement therapy), smoking, coronary artery disease, and the use of antiaggregant and anticoagulant drugs in the postoperative period were evaluated within the scope of variables related to general health status by obtaining data from the patient’s medical records.

The study protocol was approved by the Ethics Committee of Eskişehir Osmangazi University Faculty of Medicine; approval date: 9 September 2025, decision number: 07. The study was conducted in accordance with the principles of the Declaration of Helsinki. Informed consent was obtained from all patients before the operation.

Surgical Technique

Under general anesthesia, the common femoral artery was exposed through a groin incision and the above-knee popliteal artery through a medial distal thigh incision. After systemic heparinization (100 IU/kg), an appropriately sized Dacron graft was anastomosed to the common femoral artery proximally and to the above-knee popliteal artery distally using standard vascular technique.

Postoperative Period

In the postoperative period, all patients were monitored for 24 h in the Cardiovascular Surgery intensive care unit. During this process, heparin infusion was given in an appropriate dose according to weight. Appropriate antiplatelet or anticoagulant therapy was initiated on the first postoperative day.

Statistical Analysis

Patient data were obtained from follow-up examinations and data from the patient record system. After discharge, patients were followed in the outpatient clinic at 1, 3, 6, and 12 months with clinical examination and duplex ultrasonography; computed tomography angiography was performed when duplex findings or clinical symptoms suggested graft failure. The obtained data were evaluated using the SPSS (Statistical Package for the Social Sciences version 27.0 IBM Corp., Armonk, NY, USA) statistical package program. Categorical variables were given as numbers (percentages), and continuous variables were given as mean ± standard deviation or median as appropriate. For comparisons between groups, the chi-square or Fisher exact test was used for categorical variables; for continuous variables, the Student *t*-test or Mann–Whitney U test was used depending on the suitability. Statistical significance was accepted as two-sided *p* < 0.05. The primary inferential analysis treated sACR as a continuous variable. Median-based sACR groups were used only for descriptive baseline comparisons and Kaplan–Meier visualization. ROC curve analysis was performed to evaluate the discriminatory ability of sACR for 1-year reintervention. The Youden index-derived cutoff was interpreted as an exploratory, hypothesis-generating threshold rather than a validated clinical decision point. To identify predictors of 1-year reintervention, univariable and multivariable Cox proportional hazards regression analyses were performed, and results were reported as hazard ratios (HRs) with 95% confidence intervals.

## 3. Results

### 3.1. Demographic Characteristics

A total of 132 patients were included in the study. Seven of the patients were female (5.3%), and 125 were male (94.7%). The groups were similar in terms of demographics and clinical baseline characteristics. In both groups, the male gender ratio was higher, and no significant difference was detected in terms of gender distribution between the groups. The median age in both groups was found to be 71 years. There were no statistically significant differences between the groups in terms of diabetes mellitus, hypertension, coronary artery disease, and smoking habits. However, the rate of chronic kidney failure requiring hemodialysis showed a statistically significant difference between the groups. This rate was detected in 8 patients (12.1%) in the first group, while it was observed in 1 patient (1.5%) in the second group (*p* = 0.033) (Table 1).

### 3.2. Laboratory Findings

When laboratory parameters were evaluated, renal function indicators were significantly worse in the low sACR group. eGFR level was found to be 63.0 ± 18.9 mL/min/1.73 m^2^ in the low sACR group, while it was 85.5 ± 13.3 mL/min/1.73 m^2^ in the high sACR group (*p* < 0.001). Similarly, the creatinine level was significantly higher in the low sACR group [1.1 (1.0–1.3) mg/dL vs. 0.8 (0.8–1.0) mg/dL; *p* < 0.001]. The urea level was also significantly higher in the low sACR group compared to the high sACR group [19 (14–23) mg/dL vs. 16 (12–18) mg/dL; *p* = 0.002].

In terms of nutrition- and protein-metabolism-related parameters, total protein and albumin levels were significantly lower in the low sACR group. The total protein level was 6.3 ± 0.9 g/dL in the low sACR group and 6.8 ± 1.0 g/dL in the high sACR group (*p* = 0.013). Albumin levels were 3.4 (3.2–3.8) g/dL in the low sACR group and 4.3 (4.0–4.5) g/dL in the high sACR group (*p* < 0.001).

No statistically significant differences were observed between the groups for glucose, platelet count, LDL cholesterol, or hemoglobin levels. Additionally, antiplatelet and anticoagulant therapy were similar across the groups. The antiplatelet drug usage rate was 62.1% in the low sACR group and 63.6% in the high sACR group (*p* = 0.857). The rates of anticoagulant use were determined to be 51.0% and 49.0%, respectively (*p* = 0.857) (Table 1).

### 3.3. Cumulative Reintervention Rates

During the follow-up period, reintervention rates were significantly higher at all time points in the low sACR group. In the first month, the reintervention rate was 12.1% in the low sACR group, compared with 1.5% in the high sACR group (*p* = 0.033). In the third month, these rates were 18.2% and 4.5%, respectively (*p* = 0.014).

At the sixth month, the reintervention rate increased significantly in the low sACR group and reached 37.9%, while it remained at 4.5% in the high sACR group (*p* < 0.001). At the end of one-year follow-up, the reintervention rate was found to be 47.0% in the low sACR group and 7.6% in the high sACR group, and this difference was statistically significant (*p* < 0.001) (Table 1).

In general, a low preoperative sACR value was associated with poorer renal function indicators, lower serum protein and albumin levels, and significantly higher cumulative reintervention rates during follow-up.

### 3.4. Clinical Outcomes

Of the 132 patients included in the study, 36 patients underwent embolectomy due to graft occlusion. Among these patients, five required repeat embolectomy due to recurrent graft occlusion.

In total, major below-knee amputation was performed on 4 patients. Two of these patients were in the group that underwent surgery a second time due to graft occlusion. During the follow-up period, all-cause mortality occurred in 32 patients.

### 3.5. ROC Analysis for Prediction of 1-Year Reintervention

**Figure 1:**
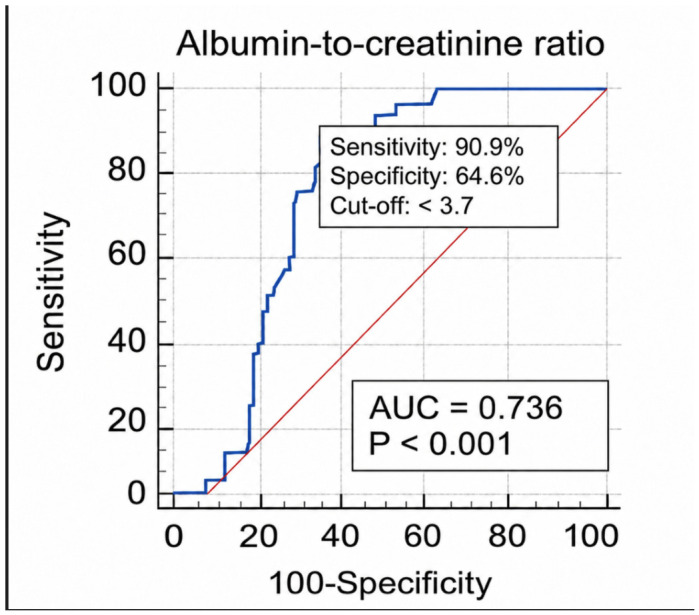
Receiver operating characteristic curve of the serum albumin-to-creatinine ratio for predicting 1-year reintervention. The blue line represents the ROC curve, whereas the red diagonal line represents the reference line of no discrimination. The area under the curve was 0.736 (95% CI: 0.634–0.838; *p* < 0.001). The optimal cutoff value of <3.7 yielded 90.9% sensitivity, 64.6% specificity, 49.1% positive predictive value, and 95.0% negative predictive value.

The performance of the albumin-to-creatinine ratio in predicting the development of a reintervention within 1 year was evaluated using ROC analysis. The area under the ROC curve (AUC) was 0.736 (95% CI: 0.634–0.838; *p* < 0.001). In exploratory ROC analysis, the Youden index suggested a cutoff value of ≤3.7, which was close to the median sACR value used for descriptive grouping. For this cutoff value, the sensitivity was 90.9%, and specificity was 64.6%. For this cutoff value, the positive predictive value was approximately 49.1%, and the negative predictive value was approximately 95.0% (Figure 1).

### 3.6. Cox Proportional Hazards Regression Analysis for 1-Year Reintervention

Importantly, sACR was entered into the Cox proportional hazards regression model as a continuous variable rather than as a dichotomized ROC-derived cutoff. Given the limited number of events, a parsimonious multivariable Cox model was constructed by including clinically relevant variables and variables associated with the outcome in univariable analysis. To minimize potential multicollinearity, variables showing strong biological or mathematical overlap were not simultaneously retained in the final multivariable model. Variables were selected according to clinical relevance, univariable analysis results, and avoidance of redundancy among closely related clinical or laboratory parameters.

In the univariate Cox proportional hazards regression analysis to predict one-year reintervention, the serum albumin/creatinine ratio was significantly associated with reintervention. It was found that an increase in the albumin/creatinine ratio was associated with a decrease in the risk of reintervention during follow–up (HR: 0.56; 95% CI: 0.41–0.78; *p* < 0.001). Similarly, the total protein level was significantly associated with the 1–year risk of reintervention in the univariate analysis (HR: 0.53; 95% CI: 0.40–0.71; *p* < 0.001). Although the platelet count showed borderline significance for reintervention, it did not reach statistical significance (HR: 0.996; 95% CI: 0.992–1.000; *p* = 0.070). There was no significant association between age, diabetes mellitus, hypertension, smoking, hemoglobin level, LDL cholesterol, glomerular filtration rate, postoperative anticoagulant use, and postoperative antiaggregant use and 1-year reintervention development.

In the multivariate Cox proportional hazards regression analysis, the albumin/creatinine ratio remained an independent predictor of 1–year reintervention even after adjustment for other variables included in the model (HR: 0.61; 95% CI: 0.43–0.87; *p* = 0.007). The total protein level was also independently associated with a decreased risk of reintervention in the multivariate analysis (HR: 0.59; 95% CI: 0.43–0.81; *p* < 0.001). In contrast, platelet count was not an independent predictor of reintervention in the multivariate model (HR: 1.00; 95% CI: 0.996–1.004; *p* = 0.840) (Table 2). In general, these findings indicate that a low albumin/creatinine ratio and low total protein levels are independently associated with an increased risk of developing reintervention at 1 year.

In general, these findings indicate that low albumin/creatinine ratio and low total protein levels are independently associated with an increased risk of developing reintervention at 1 year.

### 3.7. Kaplan–Meier Analysis

**Figure 2:**
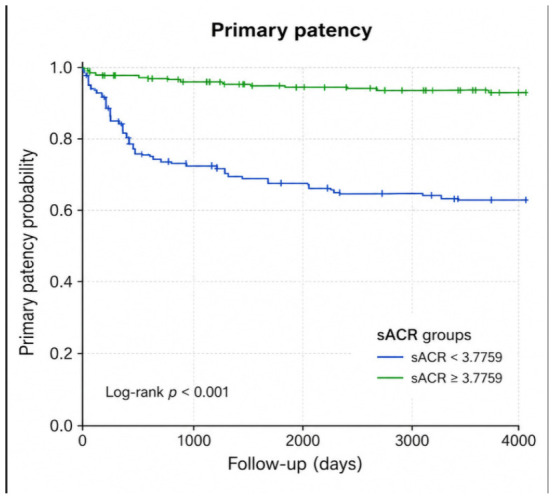
Kaplan–Meier curve for primary patency according to sACR groups. Primary patency was significantly lower in patients with sACR < 3.7759 compared with those with sACR ≥ 3.7759 (log-rank *p* < 0.001).

In the Kaplan–Meier analysis, sACR groups were compared with respect to primary patency. These groups were based on the median preoperative sACR value and were used for graphical visualization rather than for defining a validated clinical cutoff. In patients with sACR < 3.7759, the primary patency was lower during follow-up, as indicated, whereas in patients with sACR ≥ 3.7759, the primary patency was preserved at a higher rate. In the log-rank analysis, the difference between the groups was statistically significant (*p* < 0.001). These findings indicate that low sACR levels are associated with reduced primary patency and earlier loss of graft patency (Figure 2).

**Figure 3:**
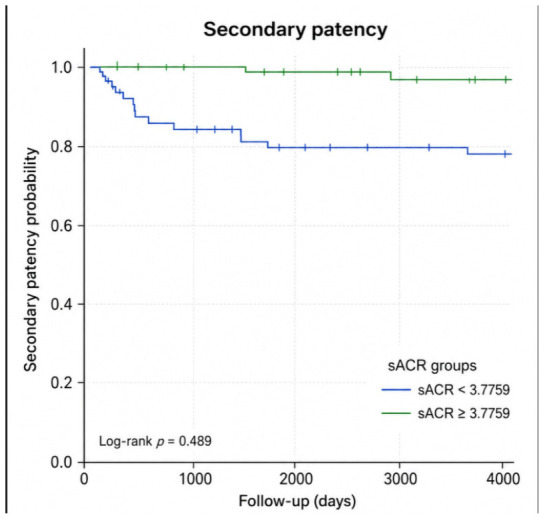
Kaplan–Meier curve for secondary patency according to sACR groups. No significant difference was observed between the two groups (log-rank *p* = 0.489).

Kaplan–Meier analysis showed no statistically significant difference in secondary patency between the low- and high-sACR groups during follow-up (log-rank *p* = 0.489). (Figure 3).

**Figure 4:**
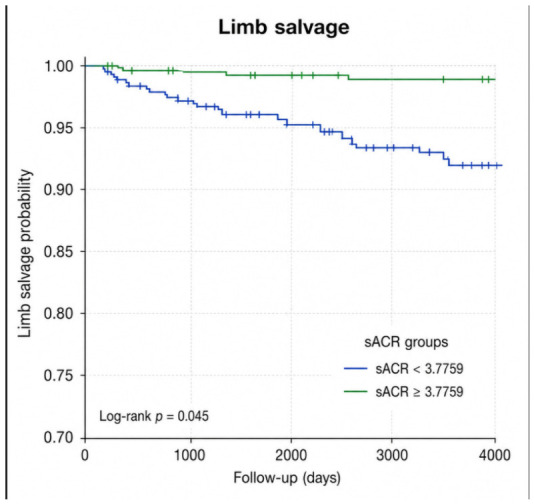
Kaplan–Meier curve for limb salvage according to sACR groups. Limb salvage was significantly lower in patients with sACR < 3.7759 compared with those with sACR ≥ 3.7759 (log-rank *p* = 0.045).

In the Kaplan–Meier analysis based on limb salvage descriptions, patients with sACR < 3.7759 had lower limb salvage probability during the follow-up period, whereas limb salvage rates remained higher in patients with sACR ≥ 3.7759. In the log-rank analysis, the difference between the groups was statistically significant (*p* = 0.045). This finding suggests that low sACR levels may be associated with an increased risk of developing major amputation (Figure 4).

**Figure 5:**
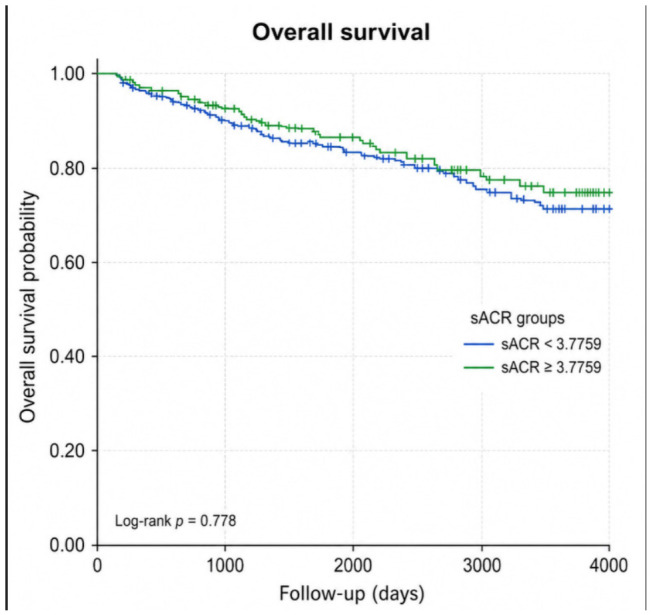
Kaplan–Meier curve for overall survival according to sACR groups. No significant difference in overall survival was observed between the two groups (log-rank *p* = 0.778).

In the Kaplan–Meier analysis based on the overall survival description, the survival curves for the sACR < 3.7759 and sACR ≥ 3.7759 groups appeared similar during the follow-up period; no statistically significant difference was found between the groups (log-rank *p* = 0.778). This finding indicates that sACR level was not significantly associated with overall survival in this cohort (Figure 5).

Since the freedom from reintervention corresponded to the same event time as the primary patency loss, the Kaplan–Meier curve showed a similar pattern to the primary patency curve. Therefore, freedom from reintervention findings were interpreted together with the primary patency results.

## 4. Discussion

Current PAD guidelines emphasize that prognosis is influenced not only by lesion anatomy but also by cardiovascular risk burden, comorbidities, functional reserve, and nutritional or inflammatory status [3,12]. In this context, sACR may be a more holistic reflection of multiple pathophysiological pathways that can trigger clinical deterioration in peripheral artery disease by combining the “nutrition–inflammation axis” (albumin) and the “renal reserve/comorbidity burden” (creatinine) into a single ratio. Although the serum albumin-to-creatinine ratio may reflect nutritional and inflammatory status, it should not be interpreted solely as a nutritional marker. Since creatinine is influenced by renal function and muscle mass, sACR may also reflect underlying renal dysfunction or systemic frailty.

Therefore, the prognostic association observed in this study should be interpreted as a composite reflection of nutritional, inflammatory, renal, and overall clinical status. Our findings regarding the prognostic value of sACR are consistent with current studies published in different cardiovascular populations. In patients undergoing percutaneous coronary intervention due to ST-elevation myocardial infarction, low sACR has been reported to be associated with adverse short-term clinical outcomes [11]. Similarly, secondary analysis based on Dryad databases has shown that low sACR may be associated with increased risk in terms of clinical outcomes [13]. Prospective cohort data reporting that sACR after percutaneous coronary intervention in very elderly patients with acute coronary syndrome can predict 1-year mortality supports the usability of this ratio in risk stratification [14]. There is also data indicating that sACR is inversely associated with short- and long-term mortality in aortic pathologies [15]. This literature suggests that sACR may be a common indicator of “systemic fragility” in different vascular beds and different clinical situations.

Albumin level is a biomarker associated with inflammation, oxidative stress, endothelial function, and catabolic status, providing indirect information about “disease severity” and “reserve” in many clinical contexts [16]. A recent study in a peripheral artery disease population found that low albumin levels were associated with increased morbidity and mortality after open and endovascular lower extremity interventions, supporting the clinical importance of albumin in predicting vascular surgical outcomes [9]. In our study, the higher incidence of events in the group with low sACR indicates that the risk may be increased by the addition of decreased renal reserve to the adverse effects of albumin (malnutrition/inflammation, oxidative stress, and pro-thrombotic tendency).

Serum creatinine is one of the key indicators of renal function, and the incidence and clinical severity of PAD increases in the presence of chronic kidney disease (CKD); vascular healing is negatively affected by mechanisms such as endothelial dysfunction, accelerated atherosclerosis, uremic toxin load, and chronic inflammation [5]. Current review/consensus texts on the management and risk reduction of CKD-PAD comorbidities emphasize that adverse cardiovascular and extremity outcomes are prominent in this patient group and that risk reduction strategies should be individualized [6]. In this context, the “creatinine component” of sACR can contribute to identifying patients who may have a worse clinical course after revascularization in the early stages by reflecting renal reserve and comorbidity burden.

A low sACR can represent a risk profile that combines the catabolic/inflammatory background accompanying hypoalbuminemia on the one hand, and the vascular fragility due to renal dysfunction on the other, which can be decisive on graft patency and clinical outcomes. Graft thrombosis is not only caused by local technical factors; it is a multifactorial process associated with multiple mechanisms such as hemodynamic overload, wall mechanics, intimal hyperplasia, and systemic inflammation [17]. Therefore, it seems biologically plausible that a marker summarizing the systemic status, such as sACR, indirectly reflects the biological infrastructure affecting early thrombosis/restenosis tendency or late patency. From a clinical application perspective, the most important advantage of sACR is that it can be calculated from two parameters already routinely measured (albumin and creatinine) without incurring additional costs. In this way, it can be integrated into preoperative risk assessment and postoperative follow-up strategies (closer clinical-radiological follow-up, comorbidity optimization, nutrition/inflammation management). In addition, current studies comparing antithrombotic strategies in the peripheral artery disease patient population operated on using synthetic grafts have emphasized the need for individualization of results according to the patient-based risk profile [18,19]. sACR can offer a simple additional layer that can help identify the high-risk patient group in this individualization approach. Although sACR showed acceptable sensitivity and a high negative predictive value, its modest specificity and limited positive predictive value indicate that it should not be used as a stand-alone decision-making tool. Rather, sACR may serve as an adjunctive screening marker to identify patients who may benefit from closer postoperative surveillance.

An important methodological consideration is that the median-based sACR grouping and the ROC-derived cutoff served different purposes in this study. The median-based grouping was used only to facilitate descriptive comparisons and Kaplan–Meier visualization, whereas the primary inferential analysis evaluated sACR as a continuous variable. The ROC-derived cutoff was not used to define the primary study groups and should not be interpreted as a validated clinical threshold. Rather, it should be considered an exploratory, hypothesis-generating value that requires external validation in independent cohorts.

Our study has some limitations. The main limitation of the present study is the absence of detailed anatomical lesion characteristics and distal runoff-related variables. These parameters are well-established determinants of bypass graft patency in vascular surgery. Due to the retrospective design, TASC classification, lesion length, degree of calcification, distal target vessel quality, and standardized runoff scoring were not consistently available for all patients. Therefore, these variables could not be included in the multivariable analysis. This limitation should be considered when interpreting the prognostic value of sACR. The retrospective single-center design and exclusion of patients without regular follow-up may have introduced selection bias, potentially excluding patients with poor outcomes or limited access to care. Second, the sample size is limited to 132 patients, and the low number of female patients in particular may limit the generalizability of the results to the entire PAD population. Third, only patients undergoing prosthetic above-knee femoropopliteal bypass with Dacron grafts were included; therefore, the findings may not be generalizable to vein grafts, PTFE grafts, or other revascularization strategies.

Inflammatory biomarkers such as C-reactive protein and procalcitonin, as well as formal nutritional assessment tools, were not routinely available; therefore, the relative contributions of inflammation and malnutrition to low sACR could not be distinguished. In addition, detailed anatomical severity variables, including runoff status, lesion length, and standardized duplex velocity criteria, were not available for all patients. Finally, although sACR demonstrated high sensitivity and negative predictive value, its moderate specificity and limited positive predictive value suggest that it should be considered an adjunctive risk marker rather than a stand-alone clinical decision tool.

Another limitation is that the ROC-derived cutoff was obtained from the same retrospective cohort and was not externally validated. Therefore, although this cutoff may be useful for exploratory risk stratification, it should not be considered a definitive clinical decision threshold. Despite these limitations, the study demonstrates the potential value of sACR in predicting reintervention in patients undergoing femoropopliteal bypass for PAD and provides a strong foundation for future prospective, multicenter research.

## 5. Conclusions

Our findings suggest that preoperative sACR is independently associated with 1-year reintervention after prosthetic above-knee femoropopliteal bypass for PAD. As a readily obtainable, low-cost, and practical parameter from routine biochemical tests, sACR may be clinically useful as an adjunctive marker for risk stratification and planning of close monitoring strategies after femoropopliteal bypass surgery. Nevertheless, the ROC-derived cutoff should be interpreted as exploratory, and these findings should be validated in larger prospective multicenter studies and further examined in relation to graft patency and limb-related outcomes. However, these findings should be considered preliminary and hypothesis-generating rather than practice-changing, and should be validated in prospective studies including anatomical and runoff-related variables.

## Figures and Tables

**Table 1:** Baseline characteristics, laboratory parameters, and cumulative reintervention rates according to preoperative serum albumin-to-creatinine ratio groups.

Variable	sACR < 3.7759 (*n*= 66)	sACR ≥ 3.7759 (*n* = 66)	*p*-Value
Male sex, *n* (%)	62 (93.9)	63 (95.5)	1.000
Age, years, median [IQR]	71 (67–76)	71 (65–75)	0.499
Diabetes mellitus, *n* (%)	23 (34.8)	26 (39.4)	0.589
Hypertension, *n* (%)	32 (48.5)	31 (47.0)	0.862
Current smoking, *n* (%)	18 (27.3)	18 (27.3)	1.000
Hemodialysis-dependent chronic kidney disease, *n* (%)	8 (12.1)	1 (1.5)	0.033
Coronary Artery Disease, *n* (%)	27 (40.9)	26 (39.4)	0.859
eGFR (mL/min/1.73 m^2^)	63.0 ± 18.9	85.5 ± 13.3	<0.001
Platelet count (×10^3^/µL), mean ± SD	234.8 ± 81.0	253.6 ± 85.6	0.199
Total protein (g/dL)	6.3 ± 0.9	6.8 ± 1.0	0.013
Reintervention within 1 month, *n* (%)	8 (12.1)	1 (1.5)	0.033
Reintervention within 3 months, *n* (%)	12 (18.2)	3 (4.5)	0.014
Reintervention within 6 months, *n* (%)	25 (37.9)	3 (4.5)	<0.001
Reintervention within 1 year, *n* (%)	31 (47.0)	5 (7.6)	<0.001
Glucose (mg/dL), median [IQR]	134 (96–189)	115 (95–196)	0.684
Urea (mg/dL), median [IQR]	19 (14–23)	16 (12–18)	0.002
Albumin (g/dL), median [IQR]	3.4 (3.2–3.8)	4.3 (4.0–4.5)	<0.001
Creatinine (mg/dL), median [IQR]	1.1 (1.0–1.3)	0.8 (0.8–1.0)	<0.001
LDL cholesterol (mg/dL), median [IQR]	116 (97–138)	132 (102–162)	0.086
Hemoglobin (g/dL), mean ± SD	13.4 ± 2.0	13.5 ± 2.1	0.768
Antiaggregant use	41 (62.1%)	42 (63.6%)	0.857
Anticoagulant use	25 (51.0%)	24 (49%)	0.857

Abbreviations and note: sACR, serum albumin-to-creatinine ratio; IQR, interquartile range; SD, standard deviation; LDL, low-density lipoprotein. Note: Reintervention was defined as reintervention for symptomatic graft occlusion confirmed by duplex ultrasonography and/or computed tomography angiography. Values at 1, 3, 6, and 12 months are cumulative.

**Table 2:** Univariable and multivariable Cox proportional hazards regression analyses for 1-year reintervention.

Baseline Characteristic	Univariable HR (95% CI)	*p* Value	Multivariable HR (95% CI)	*p* Value
Serum Albumin to creatinine ratio	0.56 (0.41–0.78)	<0.001	0.61 (0.43–0.87)	0.007
Age	0.98 (0.94–1.02)	0.260	—	—
Diabetes mellitus	1.13 (0.56–2.27)	0.732	—	—
Hypertension	1.28 (0.65–2.50)	0.474	—	—
Smoking	0.67 (0.33–1.34)	0.252	—	—
Hemoglobin	0.99 (0.84–1.16)	0.851	—	—
LDL cholesterol	0.995 (0.987–1.003)	0.211	—	—
Postoperative anticoagulant use	0.65 (0.34–1.27)	0.208	—	—
Postoperative antiplatelet use	0.65 (0.34–1.27)	0.208	—	—
eGFR	0.996 (0.98–1.01)	0.674	—	—
Platelet count (PLT) (×10^3^/µL)	0.996 (0.992–1.000)	0.070	1.00 (0.996–1.004)	0.840
Total protein (g/dL)	0.53 (0.40–0.71)	<0.001	0.59 (0.43–0.81)	<0.001

Abbreviations: HR, hazard ratio; CI, confidence interval; GFR, glomerular filtration rate; PLT, platelet count.

## Data Availability

The data presented in this study are available from the corresponding author on reasonable request.
